# Cybersickness Variability by Race: Findings From 6 Studies and a Mini Meta-analysis

**DOI:** 10.2196/36843

**Published:** 2022-06-01

**Authors:** Alison Jane Martingano, Ellenor Brown, Sydney H Telaak, Alexander P Dolwick, Susan Persky

**Affiliations:** 1 Social and Behavioral Research Branch National Human Genome Research Institute National Institutes of Health Bethesda, MD United States; 2 Office of Science and Engineering Laboratories US Food and Drug Administration Silver Spring, MD United States

**Keywords:** cybersickness, racial differences, virtual reality, head-mounted displays, simulator sickness

## Abstract

**Background:**

With the influx of medical virtual reality (VR) technologies, cybersickness has transitioned from a nuisance experienced during leisure activities to a potential safety and efficacy concern for patients and clinicians. To improve health equity, it is important to understand any potential differences in cybersickness propensity among demographic groups, including racial groups.

**Objective:**

This study aims to explore whether cybersickness propensity differs across racial groups.

**Methods:**

We collected self-reported cybersickness ratings from 6 racially diverse independent samples within 1 laboratory group (N=931). In these studies, the participants were asked to perform tasks in VR such as traversing environments, pointing at and selecting objects, and interacting with virtual humans.

**Results:**

Significant racial differences in cybersickness were found in 50% (3/6) of studies. A mini meta-analysis revealed that, on average, Black participants reported approximately one-third of SD less cybersickness than White participants (Cohen *d*=−0.31; *P*<.001), regardless of the nature of the VR experience. There was no overall difference in reported cybersickness between the Asian and White participants (Cohen *d*=−0.11; *P*=.51).

**Conclusions:**

Racial differences in cybersickness indicate that researchers, practitioners, and regulators should consider patient demographics when evaluating VR health intervention outcomes. These findings lay the groundwork for future studies that may explore racial differences in cybersickness directly.

## Introduction

### Background

Cybersickness is a common negative physiological effect of exposure to virtual reality (VR), with symptoms similar to motion sickness, including disorientation, nausea, headache, and eye strain [1]. Recent technological advances have led to the widespread use of low-cost VR technologies. In turn, VR technologies that were developed and primarily used for gaming and entertainment have been applied broadly across areas such as education, industry, and medicine. Medicine, in particular, is a rapidly expanding application space for VR technologies, including new developments in a range of areas such as medical education and training [2], physical therapy and rehabilitation [3], surgical planning [4], pain management [5-7], psychotherapy [8,9], and treatment for ophthalmic disorders [10]. As medical VR technologies have become more commonplace, cybersickness has transitioned from a nuisance to a potential safety and efficacy concern. Cybersickness concerns have prompted researchers [11-14], professional groups [15], standards organizations [16], and the US Food and Drug Administration [17] to address prevention, assessment, and mitigation strategies.

When researchers and clinicians are making benefit-risk determinations for emerging VR technologies, they should consider cybersickness propensity. Although cybersickness is a well-known response to VR exposure, the full range of causes and risk factors are not well understood. This knowledge gap may be a barrier in assessing the safety and effectiveness of VR technologies for all users. Thus far, the scientific literature has identified several factors that are linked to differences in cybersickness risk. It is well known that elements of VR content, VR hardware, and the interface between them influence cybersickness outcomes [18,19]. Certain demographic and within-person factors have also been connected with propensity to experience cybersickness, such as age [18,20-23], sex and gender [18,22,24-26], BMI [27], and health and health history [28-30]. A large meta-analysis of existing literature (*k*=137) recently found that various individual differences predict cybersickness propensity, including gender, real-world experience, technological experience, possessing a neurological disorder, and possessing a relevant phobia [31]. However, this meta-analysis did not consider race as a potential moderator.

### Racial Differences in Cybersickness and Motion Sickness

Studies investigating potential cybersickness differences by user race could provide valuable new insights into potential inequities in VR accessibility, which is critical for ensuring that this emerging technology is accessible to all in the future. Currently, such studies are lacking in the cybersickness literature. However, early research has suggested that there are racial differences in motion sickness propensity. For example, a series of studies conducted in the United States found that Asian participants reported more motion sickness symptoms than White and Black participants [32-34]. This racial difference was maintained regardless of whether the Asian participants were born in the United States or were recent immigrants [35]. This led the authors to posit an evolutionary and genetic basis for these differences. However, this conclusion is at odds with the modern understanding that race is a social construct rather than a biological or genetic one. As such, the identified differences in motion sickness reporting by race may alternatively reflect cultural and social differences that result, in part, from systemic differential treatment. Various other sociocultural factors may contribute to racial differences in reporting of discomfort such as language, acculturation, learning and cultural conditioning, and attention to uncomfortable stimuli (refer to Lasch [36]). More recent research conducted in Germany found that Asian participants reported less motion sickness than White participants [37]. However, this study found that Asian participants had a shorter tolerance for rotation despite reporting less motion sickness, which may indicate differences in motion sickness reporting that are separate from physical experience. Overall, the existing research on racial differences in motion sickness is limited. Moreover, cybersickness, although related to motion sickness and simulator sickness, is a distinct phenomenon, with disorientation being more common and oculomotor symptoms being less common [38]. Given these differences between motion sickness and cybersickness, racial variability in cybersickness warrants investigation.

In anticipation of evaluating VR-based medical product efficacy alongside VR-associated risks across patient demographics, it is important to understand any potential underlying differences between groups related to these outcomes. Addressing this knowledge gap aligns with an increasing regulatory focus on health equity [39] as well as related efforts to promote diversity in study populations and evaluate potential differential product outcomes by patient demographics [40]. The potential for race-related variability in the performance of medical technologies was recently illustrated by a safety communication on the limitations of pulse oximeter devices, which highlighted the potential accuracy differences between patients with dark and light skin pigmentation [41]. Similarly, medical use of VR may be susceptible to racial inequities in ways that have not yet been uncovered. Thus, although theory and previous literature do not provide a clear path toward hypothesizing racial differences in cybersickness, it is important to explore existing data associated with VR use to determine whether racial variability in cybersickness exists. Understanding the differences in cybersickness propensity based on race is critical to ensuring that this emerging technology is accessible to all in the future.

Currently, studies exploring racial differences in cybersickness are lacking. To address this gap in the literature, we reported data from 6 independent samples collected within 1 laboratory group. In these studies, participants were asked to perform various tasks in VR such as traversing environments, pointing at and selecting objects, and interacting with virtual humans. The analyses compared self-identified Black and Asian participants’ reporting of cybersickness to that of self-identified White participants. Comparisons between Black and Asian participants were also included in individual studies, where feasible. These 3 racial groups were chosen for comparison because they were well represented across all study samples and represent groups of interest for potential disparities when evaluating VR health care devices for use in the United States. White participants were chosen as the comparison group in the analyses because they are the most represented racial group in the existing literature. We also report a mini meta-analysis to illustrate the overall trends across all 6 studies. Together these studies are intended to reveal any differences in reported cybersickness between racial groups and lay the groundwork for future studies that may explore these differences directly. To the best of our knowledge, this is the first report of racial differences in cybersickness in the literature and is therefore a critical first step that should be explored in future research. Ultimately, addressing racial differences in cybersickness will help to move toward greater health equity.

## Methods

### Overview

This analysis included data from 6 experimental trials conducted for other purposes (Table 1). All studies were conducted between 2009 and 2020 through the Immersive Simulation Program at the National Human Genome Research Institute, National Institutes of Health. All research participants were recruited from the local community. VR was used at the program’s laboratory facility.

Each study used one of 2 types of VR settings: a buffet restaurant environment called the VR buffet [42] or a clinical examination room environment. Both VR programs were created using the Vizard VR platform [43]. Studies were selected for inclusion because they administered measures of participant cybersickness symptoms using a variant of the Short Symptoms Checklist (SSC) [44], because they recorded participants’ self-reported race, and because data were available for analysis. In all studies, the possibility of experiencing cybersickness was communicated to participants both in the consent form and by the research assistant during the study. Participants were told that they were welcome to stop the study if they experienced any cybersickness symptoms. This rarely occurred in practice. More details about each study are available in the original publications [45-50].

**Table 1:** Characteristics of the virtual reality (VR) environment for each research study.

	Year	Content	Locomotion	Headset	Aim of the study
Study 1	2017	VR buffet	Walking	HTC Vive	Measure the influence of messages about children’s diet on parents’ feeding behavior
Study 2	2011	VR buffet	Walking	nVisor SX60	Measure the influence of children’s risk information provision on parents’ feeding behavior
Study 3	2009	Virtual clinic	Walking	nVisor SX60	Assess medical students’ reaction to a virtual patient’s weight in a clinical scenario
Study 4	2020	Virtual clinic	Seated	HTC Vive Pro	Assess medical students’ use of a virtual patient’s genomic risk information in a clinical scenario
Study 5	2014	Virtual clinic	Seated	nVisor SX60	Assess reaction of women with overweight to virtual provider’s messages
Study 6	2012	Virtual clinic	Seated	nVisor SX60	Assess reaction of women with overweight to virtual provider’s messages

### VR Environments

#### The VR Buffet

**Figure 1:**
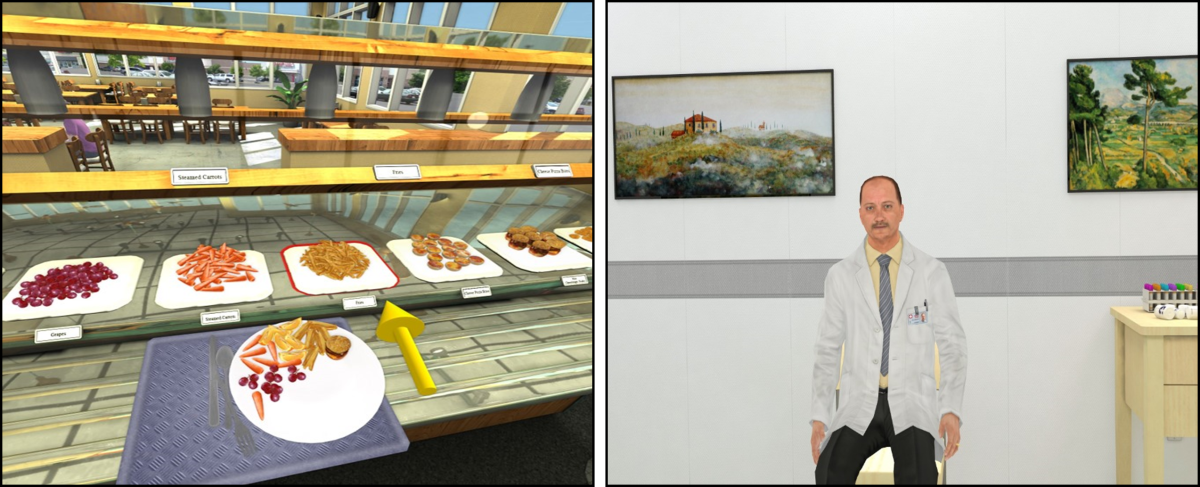
Screenshots of buffet and clinical virtual reality environments.

The VR buffet is a simulated buffet restaurant in which parental food choices for their child are assessed by tracking the parents’ virtual food selections. Outcomes for the VR buffet are a validated measure of parental food choices [42]. The participants’ physical movements drive the viewpoint in the virtual world, such that walking around the physical room corresponds to walking around the virtual buffet. Participants made food selections in the virtual buffet using a controller. Once all food and drink selections were made, participants selected a virtual cash register to indicate completion. Figure 1 shows the VR buffet environment.

#### VR Clinical Simulations

Several VR clinical simulations are included in which participants are immersed in a virtual medical examination room as either the provider or patient and asked to interact verbally with a virtual human playing the opposite role. When medical students (as opposed to patients) are the users, they are also asked to read information about the virtual patient’s medical records on a virtual computer monitor or tablet situated within the VR environment. A research assistant controlled the prerecorded statements of the virtual human interaction partner. In most cases, users are seated in this virtual environment, although there is also a version in which users can walk around and approach their virtual interaction partner. Figure 1 shows a sample VR clinical simulations.

#### VR Equipment

All studies were conducted within the same physical laboratory environment, which consisted of a room fitted with a 6-dof VR headset system. The headset and equipment used differed across studies (Table 1 provides information on the system used in each study). The earlier VR system included an NVIS nVisor SX60 headset with a WorldViz Precision Point Tracking System. A handheld presentation pointer was modified to provide hand control of the selection tool in the VR buffet environment. Later systems included an HTC Vive headset with an integrated tracking system or an HTC Vive Pro headset with an integrated tracking system. In both cases, the relevant Vive or Vive Pro controllers were used for hand control when needed.

### Study Inclusion and Exclusion Criteria

Several inclusion and exclusion criteria (eg, gender, age, and parental status) varied between studies based on the content of the specific research study. All studies also had exclusion criteria related to the use of the VR equipment. In all studies, potential participants were excluded if they reported having epilepsy, seizures, or a vestibular disorder or if they reported having vision or hearing that was neither normal nor corrected to normal. In most studies, a known pregnancy was also an exclusion criterion. Potential participants were excluded if they reported higher levels of propensity to motion sickness. Participants were asked the following question: “How easily would you say that you get motion or car sickness on a scale of 1 to 7 where 1 would be that you ‘never get motion sick’ and 7 would be that you ‘get sick easily’?” Those who answered 6 or 7 on a 7-point scale were deemed ineligible for participation. As such, all participants in the included studies were individuals who did not identify themselves as being particularly vulnerable to motion sickness symptoms.

All participants were encouraged to adjust the VR headset themselves while viewing the VR environment. For example, participants were asked to move the headset up and down on their face and then to use the adjustment that would “move the lenses closer together and further apart, allowing [them] to focus.” They were repeatedly asked whether the virtual room looked blurry and were instructed to make adjustments until they felt that their view of the room was clear. After completing the VR experience, the participants were asked to rate their level of cybersickness as part of a larger questionnaire. Demographic information including self-reported race was collected at pretest or during the laboratory visit.

### Measures

All studies included in this analysis administered a variant of the Simulator Sickness Checklist, the SSC [43]. The SSC is a commonly used self-report measure of cybersickness that contains a subset of symptoms used in the longer Simulator Sickness Questionnaire [50]. Most of the included studies used a 5-item version of the SSC that assessed headache, blurred vision, dizziness with eyes open, dizziness with eyes closed, and nausea. Clinical studies involving women with higher weight as participants (studies 5 and 6) used a 6-item version that additionally assessed eyestrain. In all studies, each item was measured on a 4-point Likert-type scale; some studies began this scale at zero, whereas others began at one. The lowest end point was labeled *none* or *not at all*, and the highest end point was labeled *severe* (Table 2). For each study, the composite cybersickness score was calculated by summing the responses for each item on the scale. In all analyses, we retained all original scale items and response options (without transformation), as these may have influenced participant responses [51]. Therefore, we caution readers that it is not possible to compare raw cybersickness scores among the included studies. We performed a mini meta-analysis for this purpose.

The primary predictor in our analysis was the race of participants included in the study. We considered the number of White, Black, and Asian participants based on each participant’s self-reported racial background. For a racial group to be considered for analysis within a given study, at least 10 participants in the study needed to self-identify with that racial group.

Additional variables assessed included self-reported gender and age. BMI was calculated from weight and height, which was self-reported except in the case of the 2 studies of medical students (studies 3 and 4), where it was measured in the laboratory. The time spent in the VR environment was automatically calculated using the VR environment software. The year of the study was determined as the year in which the last participant data collection visit occurred. Table 3 provides a summary of the demographic variables for each study.

**Table 2:** Self-reported cybersickness symptoms by racial group.

Scale	Racial group	Severity rating of cybersickness symptoms for each item, mean (SD)							Composite cybersickness, mean (SD)
		Headache	Eyestrain	Blurred vision	Dizzy (eyes open)	Dizzy (eyes closed)	Nausea		
**Study 1 (0=none, 1=slight, 2=moderate, 3=severe)^a^**									
	White	0.15 (0.44)	N/A^b^	0.48 (0.61)	0.39 (0.63)	0.09 (0.33)	0.13 (0.37)	1.23 (1.68)	
	Black	0.04 (0.20)	N/A	0.34 (0.60)	0.13 (0.45)	0.11 (0.38)	0.00 (0.00)	0.57 (1.13)	
	Asian	0.22 (0.42)	N/A	0.26 (0.45)	0.26 (0.45)	0.07 (0.27)	0.04 (0.19)	0.85 (1.10)	
	Total	0.13 (0.39)	N/A	0.40 (0.58)	0.29 (0.57)	0.09 (0.33)	0.07 (0.29)	0.98 (1.49)	
**Study 2 (0=none, 1=slight, 2=moderate, 3=severe)^a^**									
	White	0.18 (0.43)	N/A	0.44 (0.63)	0.33 (0.56)	0.19 (0.46)	0.09 (0.30)	1.23 (1.37)	
	Black	0.11 (0.32)	N/A	0.29 (0.48)	0.19 (0.39)	0.05 (0.22)	0.06 (0.24)	0.69 (0.94)	
	Total	0.15 (0.39)	N/A	0.38 (0.58)	0.27 (0.50)	0.13 (0.38)	0.08 (0.28)	1.01 (1.23)	
**Study 3 (0=none, 1=slight, 2=moderate, 3=severe)^a^**									
	White	0.12 (0.36)	N/A	0.40 (0.57)	0.36 (0.59)	0.12 (0.43)	0.14 (0.40)	1.14 (1.68)	
	Black	0.06 (0.24)	N/A	0.38 (0.55)	0.38 (0.55)	0.00 (0.00)	0.03 (0.17)	0.86 (1.05)	
	Asian	0.27 (0.54)	N/A	0.38 (0.61)	0.42 (0.68)	0.15 (0.36)	0.21 (0.46)	1.42 (2.03)	
	Total	0.15 (0.40)	N/A	0.39 (0.57)	0.38 (0.61)	0.10 (0.37)	0.14 (0.39)	1.16 (1.69)	
**Study 4 (0=none, 1=slight, 2=moderate, 3=severe)^a^**									
	White	0.12 (0.41)	N/A	1.00 (0.78)	0.12 (0.41)	0.00 (0.00)	0.00 (0.00)	1.19 (1.19)	
	Black	0.00 (0.00)	N/A	0.81 (0.91)	0.00 (0.00)	0.00 (0.00)	0.00 (0.00)	0.73 (0.88)	
	Asian	0.08 (0.28)	N/A	0.56 (0.65)	0.08 (0.28)	0.00 (0.00)	0.00 (0.00)	0.78 (0.90)	
	Total	0.08 (0.32)	N/A	0.81 (0.78)	0.08 (0.32)	0.00 (0.00)	0.00 (0.00)	0.96 (1.05)	
**Study 5 (1=not at all, 4=severe)^c^**									
	White	1.10 (0.31)	1.56 (0.66)	1.44 (0.56)	1.08 (0.27)	1.00 (0.00)	1.01 (0.11)	7.19 (1.27)	
	Black	1.03 (0.18)	1.33 (0.50)	1.36 (0.55)	1.03 (0.18)	1.03 (0.18)	1.00 (0.00)	6.81 (1.05)	
	Total	1.07 (0.25)	1.45 (0.59)	1.40 (0.56)	1.06 (0.23)	1.02 (0.13)	1.01 (0.08)	7.01 (1.18)	
**Study 6 (1=not at all, 4=severe)^c^**									
	White	1.14 (0.39)	1.36 (0.58)	1.32 (0.54)	1.03 (0.18)	1.03 (0.18)	1.02 (0.13)	6.91 (1.39)	
	Black	1.13 (0.51)	1.24 (0.49)	1.24 (0.49)	1.06 (0.28)	1.04 (0.19)	1.05 (0.25)	6.75 (1.56)	
	Total	1.13 (0.47)	1.28 (0.52)	1.27 (0.51)	1.05 (0.25)	1.04 (0.19)	1.04 (0.22)	6.81 (1.50)	

^a^Scale minimum: 0; scale maximum: 15.

^b^N/A: not applicable.

^c^Scale minimum: 6; scale maximum: 24.

**Table 3:** Demographic variables for each study.

Study	Racial group	Sample, n	Age (years), mean (SD)	Gender (female), n (%)	BMI (kg/m^2^), mean (SD)	Time in virtual reality (seconds), mean (SD)
**Study 1**						
	White	88	38.08 (5.72)	60 (68)	25.87 (5.51)	318 (323)
	Black	48	36.31 (6.35)	33 (69)	31.63 (9.32)	301 (294)
	Asian	27	39.12 (4.76)	18 (67)	25.64 (5.90)	306 (214)
**Study 2**						
	White	105	38.89 (5.33)	105 (100)	30.18 (4.78)	409 (491)
	Black	75	35.81 (5.62)	75 (100)	31.10 (5.18)	389 (452)
**Study 3**						
	White	104	26.55 (2.25)	49 (47)	23.92 (2.85)	414 (119)
	Black	34	26.56 (3.74)	21 (62)	26.08 (4.43)	433 (125)
	Asian	48	25.77 (2.48)	24 (50)	22.95 (3.74)	414 (119)
**Study 4**						
	White	34	26.41 (2.66)	20 (59)	23.26 (3.63)	664 (252)
	Black	16	26.06 (1.84)	12 (75)	25.56 (4.57)	663 (218)
	Asian	25	25.96 (1.57)	15 (60)	23.53 (4.29)	721 (185)
**Study 5**						
	White	88	35.24 (9.65)	88 (100)	31.25 (5.25)	253 (21)
	Black	85	35.55 (8.16)	85 (100)	35.55 (8.16)	255 (22)
**Study 6**						
	White	58	35.35 (8.71)	58 (100)	36.33 (7.66)	423 (60)
	Black	109	36.07 (11.24)	109 (100)	32.07 (6.03)	427 (70)

### Data Analysis

For each individual study, we conducted an ANOVA to examine the relationship between participant race (2 or 3 groups depending on whether Asian participants were present in sufficient numbers to be included) and cybersickness. When there were 3 racial groups, we examined planned contrasts to assess the differences between individual racial groups. We also examined zero-order correlations between cybersickness and 3 person-level variables: age, BMI, and time spent in the VR environment (Multimedia Appendix 1, Tables S1-S5). When these variables demonstrated significant relationships with cybersickness, they were included as covariates in an additional analysis of covariance.

We also conducted a random effects meta-analysis [52] using Comprehensive Meta-Analysis V3 software [53] to determine the overall difference in self-reported cybersickness between racial groups in our 6 studies. Analyses were performed using Cohen *d* with weighted averages of the effect sizes.

### Ethics Approval

Participants were compensated for participation in all studies, and all studies were approved by the relevant institutional review boards. IRB review approval numbers are as follows: 08HG0122, 10HG0076, 11HG0238, 13HG0125, and 16HG0026.

## Results

### Cybersickness Levels Overall

Self-reported cybersickness was very low in all racial groups (Table 2). On average, participants reported no to slight symptoms, with blurred vision and eyestrain being reported the most and nausea and dizziness (with eyes closed) being reported the least.

### Relationships Between Race and Cybersickness in Individual Studies

#### Study 1: Buffet With Parents

ANOVA revealed a significant difference in cybersickness by racial group (Table 4). Pairwise comparisons showed that Black participants reported lower levels of cybersickness than White participants. There was no significant difference between White and Asian participants. No other person-level variables showed a significant relationship with cybersickness (Multimedia Appendix 1, Table S1).

**Table 4:** Results from ANOVA within individual studies.

	Effect of racial group, omnibus analysis		Pairwise comparisons						
	*F* test (*df*)	*P* value	White vs Black				White vs Asian		
			Mean difference	*P* value	Bonferroni corrected *P* value	Mean difference		*P* value	Bonferroni corrected *P* value
Study 1	3.34 (2,157)	.03	0.84	.004	.02	0.44		.19	.38
Study 2	7.75 (1,180)	.006	N/A^a^	N/A	N/A	N/A		N/A	N/A
Study 3	1.12 (2,183)	.33	0.29	.38	.76	−0.27		.36	.72
Study 4	2.07 (2,72)	.13	0.42	.18	.84	0.52		.06	.12
Study 5	5.18 (1,173)	.02	N/A	N/A	N/A	N/A		N/A	N/A
Study 6	0.36 (1,166)	.55	N/A	N/A	N/A	N/A		N/A	N/A

^a^N/A: not applicable (pairwise comparisons are only reported for studies with more than 2 racial groups).

#### Study 2: Buffet With Mothers Only

The ANOVA revealed a significant difference in cybersickness by racial group (Table 4), wherein Black participants reported lower levels of cybersickness than White participants. There was also a significant relationship between age and cybersickness, with older participants reporting more cybersickness. However, the main effect of race on cybersickness was maintained when age was added as a covariate (*F*_1,175_=5.33; *P*=.02). No other person-level variables showed a significant relationship with cybersickness (Multimedia Appendix 1, Table S2).

#### Study 3: Clinical With Medical Students

The ANOVA did not show a significant effect of race on cybersickness. Among the person-level variables, there was a significant relationship between age and cybersickness, with older participants reporting greater cybersickness (Multimedia Appendix 1, Table S3). However, the analysis of covariance also did not show a main effect of race on cybersickness (*F*_1,180_=2.32; *P*=.44).

#### Study 4: Clinical With Medical Students

The ANOVA did not show a significant effect of race on cybersickness. No other person-level variables showed a significant relationship with cybersickness (Multimedia Appendix 1, Table S4).

#### Study 5: Clinical With Women Only

The ANOVA revealed a significant difference in cybersickness by racial group (Table 4), wherein Black participants reported lower levels of cybersickness than White participants. No other person-level variables showed a significant relationship with cybersickness (Multimedia Appendix 1, Table S5).

#### Study 6: Clinical With Women Only

The ANOVA did not show a significant effect of race on cybersickness. No other person-level variables showed a significant relationship with cybersickness (Multimedia Appendix 1, Table S6).

### Mini Meta-analysis

#### Overview

Although the results of each individual study reported above are revealing, determining whether there are racial differences in cybersickness, based on whether individual studies report a statistically significant difference, is inherently flawed. By conducting a mini meta-analysis, we were able to determine the size of these effects. In addition, by combining data in a meta-analysis, we reduced the impact of random error and increased the precision of our estimate. This increased precision allowed us to detect racial differences in cybersickness that individual studies may lack the power to detect.

**Figure 2:**
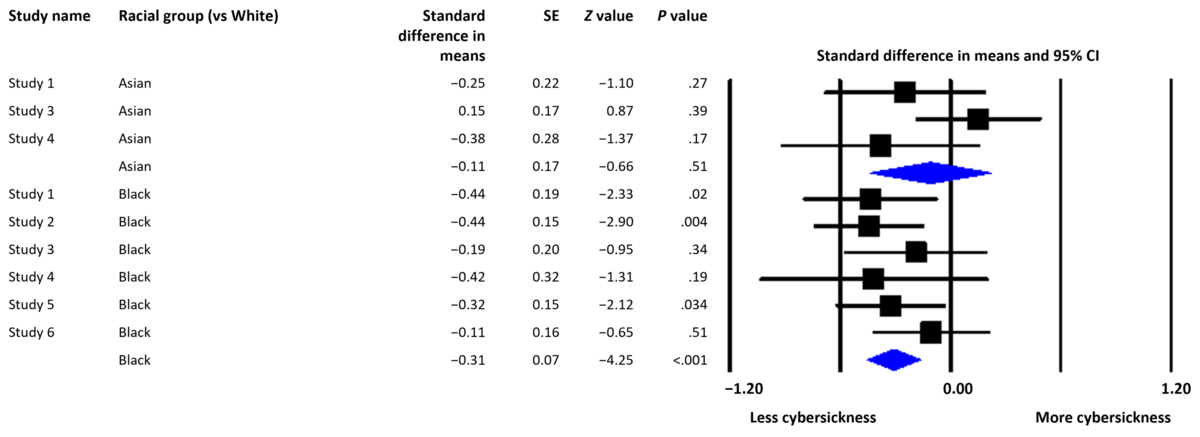
Forest plot depicting the standardized mean difference (Cohen *d*) in reported cybersickness for Black and Asian participants compared with White participants.

In our mini meta-analysis (Figure 2), effect sizes indicated the difference in reported cybersickness between White participants and Black and Asian participants. Positive effect sizes indicate that Black and Asian participants report more cybersickness than White participants, whereas negative effects indicate that Black and Asian participants report less cybersickness than White participants. Moderator analyses were conducted using mixed-effect models.

Overall, Black participants reported significantly less cybersickness than White participants (Cohen *d*=−0.31; *P*<.001; κ=6; Figure 2). On average, Black participants reported approximately one-third of an SD less cybersickness than White participants. Asian participants did not report significantly different cybersickness levels compared with White participants (Cohen *d*=−0.11; *P*=.51; κ=3; Figure 2).

#### Moderator Analyses

To explore whether the racial differences in reported cybersickness between Black and White participants may be exaggerated or attenuated in certain situations, we conducted exploratory moderator analyses. Specifically, we evaluated the VR environment (buffet vs clinical), movement (seated vs standing), headset type (nVisor SX60 vs Vive), duration of experience, and year of data collection. Asian participants were excluded from these moderator analyses. This exclusion was a conservative approach to ensure that White participants (the comparison group) were included only once in the analysis. This approach prevented artificial inflation of N and the overestimation of the precision of the effect.

The moderator analyses did not reveal any variables that attenuated racial differences in cybersickness. Black participants reported less cybersickness than White participants regardless of the nature of the VR experience. Specifically, the magnitude of the racial difference was not significantly different based on whether participants engaged with the VR buffet or the clinical VR scenario (*Q*_1_=2.155; *P*=.14). Racial differences were also unchanged regardless of whether the participants were seated or standing (*Q*_1_=0.79; *P*=.37). Racial differences in cybersickness were also consistent regardless of the type of headset (*Q*_1_=0.700; *P*=.40) and duration of the VR experience (B=−0.0001; 95% CI −0.002 to 0.002; *Z*=−0.14; *P*=.88). In addition, the year of study did not moderate racial differences in reported cybersickness (B=−0.0195, 95% CI −0.068 to 0.030; *Z*=−0.78; *P*=.44).

Among the studies included in this analysis, racial differences in cybersickness appear robust and consistent across various VR experiences and experimental designs. Overall, Black participants reported less cybersickness than White participants regardless of the nature of the VR experience.

## Discussion

### Principal Findings

This study presents the first known examination of racial differences in cybersickness. We found that, on average, Black participants reported less cybersickness than White participants, and our analyses did not reveal any moderators that attenuated this racial difference. In contrast to previous research on motion sickness [32-35,37], we found no differences in reported cybersickness between White and Asian participants. However, this comparison should be interpreted with caution, as cybersickness differs from other forms of motion sickness [38] and as we excluded potential participants who reported that they were particularly prone to motion sickness. Although our results require replication, they indicate that researchers, practitioners, and regulators may need to consider the potential for racial differences in cybersickness when evaluating VR applications for their side effects.

### Potential Explanations for Racial Differences in Cybersickness

The data reported here do not allow us to determine why different racial groups reported varying levels of cybersickness. There are a multitude of factors that could influence pathways through which individuals differ in their propensity for and reporting of cybersickness. Some of these factors are discussed below. Although our data do not support any individual causal mechanism, we highlight where theories are consistent or inconsistent with our findings. Importantly, it is likely that multiple causal forces influence cybersickness experience and reporting simultaneously, working together or in opposition with one another. Further research is needed to determine the possible causal mechanisms behind racial differences in cybersickness.

One reason that people differ in their propensity for cybersickness is their previous experience with VR [54,55]. Previous research has found that people who have never used VR report more cybersickness than people who rarely use VR, and both groups report more cybersickness than those who use VR weekly [56]. Familiarity with VR is therefore associated with lower levels of cybersickness. There is no reason to believe that familiarity with VR explains the results of our studies, as most of the research was conducted before VR became a common consumer device, and we saw no evidence that racial differences in cybersickness were attenuated or augmented in more recent years. Nevertheless, familiarity with VR technology may be a piece of this puzzle going forward. A recent survey of British internet users found that people of color are overrepresented in the VR consumer market [57]. This is supported by market research findings that Black and Hispanic consumers are more aware of and interested in VR than White consumers [58]. Therefore, familiarity-related method equivalence [59] should be monitored in future VR studies.

Another reason people differ in their propensity for cybersickness is differences in body size and proportions. Oculomotor cybersickness has been shown to decrease with higher BMI [27]. However, in these studies, racial differences largely remained when BMI was entered as a covariate, with the exception of study 1 (Multimedia Appendix 1, Table S7). Another potential factor is participants’ interpupillary distance (IPD) and goodness of headset fit. Previous research has shown that people with an IPD that is poorly accommodated by standard VR headsets are more likely to experience cybersickness [14] and that the inability to accommodate diverse bodies may lead to apparent demographic differences in cybersickness. In 2 studies, researchers demonstrated that women, for whom a VR headset built for men’s IPD specifications did not fit properly, reported higher cybersickness than men. However, when women’s headsets were fitted to them correctly, they experienced cybersickness at a rate similar to that of men [14]. As we did not measure the IPD of our participants or the goodness of headset fit, we cannot rule out the possibility of variation within our sample and thus cannot explore whether these are a potential explanation for our results. These limitations should be addressed in future research. Previous research has suggested that in some cases, self-reported racial identity is associated with differences in average IPD measurements (refer to the study by Dodgson [60]); however, it is unknown how this might influence the fit of VR headsets or subsequent cybersickness.

Individuals may also differ in their likelihood of reporting cybersickness, just as there are individual differences in reporting other types of pain and discomfort. With some exceptions, people of color generally report higher levels of pain than White patients [61-65] (refer to the study by Plesh et al [66] for contrasting results). Systemic barriers to adequate pain management and a long history of discrimination and dehumanization likely explain these trends [67]. Various other cultural factors may also contribute to differences in reporting, including language, acculturation, learning and cultural conditioning, degree of expressiveness, heightened attention to painful stimuli, and coping styles (refer to the study by Booker [61]). Given that our results contradict much of the existing research on pain and discomfort, further research is needed to understand how sociocultural factors may specifically influence the reporting of cybersickness.

Another factor relevant to cybersickness reporting is the individual differences in how people respond to questionnaires, particularly Likert-type scales. Scales that range from 1=strongly disagree to 5=strongly agree or from 1=poor to 5=excellent are often used in health research but are troublesome because responses are influenced not only by the content of the question but also by general approaches to answering such questions. Researchers have documented that certain response styles are more common among specific racial and ethnic groups. For example, Black and Latino study participants are especially likely to use the extreme positive end of rating scales [59], whereas East Asian participants are more likely to select scale midpoints and avoid extreme responses compared with North American participants [68,69]. It has been hypothesized that these response styles reflect various cultural values on which people of color generally differ from White participants [59]. In particular, manifestations of social desirability may differ across cultures [70] in ways that result in different response styles [59]. It is difficult to know how such response styles would manifest on the cybersickness scale used in this study, which ranges from *none* to *severe*. Nevertheless, it is certainly possible that racial differences in response styles may explain the differences in cybersickness reporting between Black and White participants in our research. Future research would benefit from using more objective, passive physiological approaches to measuring cybersickness such as electroencephalography [71] to overcome difficulties with self-reporting. Nevertheless, it is important to understand potential racial differences in self-reported cybersickness, for example, when evaluating novel VR medical interventions.

### Limitations

In addition to the limitations of our design that prevent us from examining why racial differences in cybersickness occur, there are other important limitations to our sample that should be considered when interpreting the results of these studies. First, we recruited participants from Washington, District of Columbia area. Participants were aware that they were volunteering for a VR experiment, and we excluded individuals who reported a high propensity for motion sickness. Therefore, the resulting samples are more likely to be interested in VR and may be less likely to experience cybersickness than the general population. In practice, exclusion because of motion sickness propensity was rare. For example, in one included sample [50], only 1.47% of potential study participants were ineligible because of this factor. Therefore, we believe that these results are a useful starting point, particularly for designing and evaluating VR medical interventions for use with populations that are not especially susceptible to cybersickness. It is worth noting that American participants represent a society that is not typical of the world’s population, which limits its representativeness [72,73]; therefore, there is no reason to expect that these same racial differences would be found outside of the US context.

Second, we excluded participants who did not identify as Asian, Black, or White from this analysis. This decision was made to ensure that we had sufficient power to detect racial differences in cybersickness. However, we were unable to draw any conclusions regarding racial groups that were not well represented in our samples. Future research should attempt to oversample people of color to achieve a sufficient sample size for other racial comparisons. Another limitation is that we excluded individuals who identified as more than one race. This may have artificially created distinct racial groups that in reality are much less coherent and discrete. In addition, we did not find many of the demographic correlations with cybersickness that have been reported in previous literature (ie, age, BMI, and time spent in VR). This is likely because of the limited range in age, BMI, and exposure time in our reported studies.

Another limitation is that we assessed cybersickness following the use of only 2 types of VR environments, neither of which is characteristic of the types of VR environments that typically elicit significant cybersickness (eg, sensory conflict and imposed motion [74]). Therefore, perhaps unsurprisingly, cybersickness ratings across all racial groups were very low. Although we anticipate that many health- and medicine-oriented VR applications will be designed to minimize cybersickness, mild cybersickness may still be of practical significance. Many medical VR experiences are designed for repeated use over time (eg, exposure therapy and pain management), and mild cybersickness may increase attrition. A recent meta-analysis of attrition in VR exposure for anxiety disorders found that attrition ranged from 2% to 41% [75]. Unfortunately, these studies rarely reported the reasons for dropout. Nevertheless, when reasons were given, cybersickness was among the top 5, accounting for 6.5% of dropouts. The most common reason given was the *inability to immerse*, which accounted for 42% of dropouts. A sense of presence is negatively related to cybersickness [19], and feeling cybersickness has been proposed as a reason for failure to immerse [76-78]. It is possible that in addition to being a direct cause of attrition itself, cybersickness may also indirectly influence attrition by reducing immersion in and enjoyment of the VR environment. Unfortunately, our study design did not allow us to investigate how cybersickness influences attrition because we used a single session of VR exposure and the discontinuation rates were very low. Research with a wider variety of VR environment types is needed, to understand how cybersickness severity relates to study attrition, intervention adherence, and the efficacy of medical VR.

### Conclusions

Here, we present data demonstrating a potentially important racial difference in cybersickness that we believe should be explored in future research. The first step in continuing this investigation would be to conceptually replicate these findings with different VR experiences, headsets, and study populations. Once replicated, research should then be conducted on the potential explanations and mechanisms. We have discussed some potential causal factors in this manuscript, but we acknowledge that there are likely other important factors that we have not considered. If future research suggests that racial differences in cybersickness are primarily in reporting as opposed to experience, it would suggest the need for the development of more objective measures of cybersickness. Such a result would also constitute an important consideration for those designing and evaluating medical VR interventions to promote health equity. Notwithstanding the caveats above, the research presented here underscores the importance of testing VR applications with a diverse group of participants to move toward achieving equitable access to emerging medical VR devices.

## Appendix

Multimedia Appendix 1 Supplementary data tables.

## Abbreviations

IPD: interpupillary distance
SSC: Short Symptoms Checklist
VR: virtual reality
