# Supplementary material for: Cybersickness Variability by Race: Findings From 6 Studies and a Mini Meta-analysis
Source: J Med Internet Res. 2022 Jun 1;24(6):e36843. doi: 10.2196/36843 (PMC9201708; doi:10.2196/36843)
Supplement: Multimedia Appendix 1 [file jmir_v24i6e36843_app1.docx]

| Table S1. Correlation Matrix showing *r* values (*P* values) for study 1 variables. | | | | | |
| --- | --- | --- | --- | --- | --- |
|  | Cybersickness | Age | Time in VR (s) | BMI |  |
| Age | -.01 (.94) |  |  |  |  |
| Time in VR (s) | -.02 (.77) | .04 (.64) |  |  |  |
| BMI | .10 (.22) | -.12 (.12) | -.02 (.80) |  |  |
| Gender | <.01 (.99) | -.08 (.32) | -.07 (.40) | -.05 (.56) |  |

| Table S2. Correlation Matrix showing *r* values (*P* values) for study 2 variables. | | | | |
| --- | --- | --- | --- | --- |
|  | Cybersickness | Age | Time in VR (s) |  |
| Age | **.16 (.031)** |  |  |  |
| Time in VR (s) | <.01 (.99) | -.01 (.94) |  |  |
| BMI | -.04 (.63) | -.04 (.63) | -.04 (.63) |  |

Note: All participants in Study 2 were female.

| Table S3. Correlation Matrix showing *r* values (*P* values) for study 3 variables. | | | | | |
| --- | --- | --- | --- | --- | --- |
|  | Cybersickness | Age | Time in VR (s) | BMI |  |
| Age | **.18 (.031)** |  |  |  |  |
| Time in VR (s) | -.05 (.57) | .06 (.48) |  |  |  |
| BMI | -.09 (.28) | .15 (.07) | -.03 (.73) |  |  |
| Gender | .16 (.05) | -.05 (.56) | -.08 (.32) | **-.31 (<.001)** |  |

| Table S4. Correlation Matrix showing *r* values (*P* values) for study 4 variables. | | | | | |
| --- | --- | --- | --- | --- | --- |
|  | Cybersickness | Age | Time in VR (s) | BMI |  |
| Age | .01 (.90) |  |  |  |  |
| Time in VR (s) | -.01 (.96) | .22 (.06) |  |  |  |
| BMI | -.17 (.16) | .05 (.66) | -.17 (.16) |  |  |
| Gender | .02 (.88) | -.05 (.65) | .02 (.88) | **.29 (.013)** |  |

| Table S5. Correlation Matrix showing *r* values (*P* values) for study 5 variables. | | | | |
| --- | --- | --- | --- | --- |
|  | Cybersickness | Age | Time in VR (s) |  |
| Age | -.01 (.95) |  |  |  |
| Time in VR (s) | .13 (.09) | .13 (.09) |  |  |
| BMI | .02 (.78) | .02 (.78) | -.07 (.34) |  |

Note: All participants in Study 5 were female.

| Table S6. Correlation Matrix showing *r* values (*P* values) for study 6 variables. | | | | |
| --- | --- | --- | --- | --- |
|  | Cybersickness | Age | Time in VR (s) |  |
| Age | -.13 (.08) |  |  |  |
| Time in VR (s) | -.03 (.69) | -.13 (.09) |  |  |
| BMI | -.12 (.13) | .15 (.06) | -.12 (.14) |  |

Note: All participants in Study 6 were female.

| Table S7. Omnibus ANCOVA for individual studies (covariates age, BMI, and time spent in VR) | |
| --- | --- |
| **Study 1** | *F*(2,148)=2.95, *P*=.055 |
| **Study 2** | *F*(1,175)=5.33, *P*=.022 |
| **Study 3** | *F*(1,180)=2.32, *P*=.44 |
| **Study 4** | *F*(2,63)=1.31, *P*=.28 |
| **Study 5** | *F*(1,168)=6.31, *P*=.013 |
| **Study 6** | *F*(1,162)=.16, *P*=.69 |
